# Roles and clinical application of exosomal circRNAs in the diagnosis and treatment of malignant tumors

**DOI:** 10.1186/s12967-022-03367-x

**Published:** 2022-04-05

**Authors:** Dong Ye, Mengdan Gong, Yongqin Deng, Shuai Fang, Yujie Cao, Yizhen Xiang, Zhisen Shen

**Affiliations:** 1 Department of Otorhinolaryngology-Head and Neck Surgery, Lihuili Hospital of Ningbo University, Ningbo, 315040 Zhejiang China; 2 Department of Thoracic Surgery, Affiliated Hospital of Ningbo University, Ningbo, 315020 Zhejiang China

**Keywords:** Exosome, CircRNAs, Malignant tumors, Function, Application

## Abstract

Exosomes are microvesicles secreted by cells. They contain a variety of bioactive substances with important roles in intercellular communication. Circular RNA (circRNA), a type of nucleic acid molecule found in exosomes, forms a covalently bonded closed loop without 5′ caps or 3′ poly(A) tails. It is structurally stable, widely distributed, and tissue specific. CircRNAs mainly act as microRNA sponges and have important regulatory roles in gene expression; they are superior to other non-coding RNAs as molecular diagnostic markers and drug treatment targets. Exosomal-derived circRNAs in the body fluids of tumor patients can modulate tumor proliferation, invasion, metastasis, and drug resistance. They can be used as effective biomarkers for early non-invasive diagnosis and prognostic evaluation of tumors, and also represent ideal targets for early precision therapeutic intervention. This review provides a theoretical basis for exploring the applications of exosomal circRNAs in malignant tumor diagnosis and treatment. We describe the biological functions of exosomal circRNAs in the occurrence and development of malignant tumors, their potential utility in diagnosis and treatment, and possible mechanisms.

## Introduction

The occurrence and development of malignant tumors are multifactorial and multi-linked intricate processes. The most important characteristic of malignant tumors is their ability to grow, infiltrate, and diffuse to surrounding tissues without restraint. The morbidity and mortality of malignant tumors are high worldwide and are increasing markedly, representing a non-negligible threat to human health. Most patients are diagnosed at an advanced stage owing to the insidious onset of the disease, which leads to deterioration of their condition and causes them to miss the best time for treatment. Although there has been some progress recently in the diagnosis and treatment of malignant tumors, early diagnosis and effective precision treatment to improve the survival rate remain major challenges in world public health and are key to precision medicine and individualized treatment of malignant tumors. At present, early diagnosis methods and effective treatments are very limited, and the mechanisms of occurrence and progression have not yet been clearly specified. Therefore, it is crucial to explore new non-invasive biomarkers for early diagnosis and treatment of malignant tumors and apply them in clinical practice.

Exosomes, which have an average diameter of 30–150 nm and a double-layered membrane structure, are extracellular vesicles secreted by a variety of cells by exocytosis. Exosomes are important carriers for signaling between cells and are present in body fluids such as saliva, blood, urine, cerebrospinal fluid, and breast milk. Their diverse constituents include almost the entire cell-derived molecular spectrum: circular RNA (circRNA), DNA, mRNA, microRNA (miRNA), proteins, and lipids [1]. Under physiological or pathological conditions, most cells can promote tumor occurrence, development, and metastasis by releasing exosomes containing the above substances into the microenvironment and transferring materials and information between cells.

CircRNAs are important non-coding RNA molecules (ncRNAs) that are produced by alternative splicing of premature mRNAs and usually do not encode proteins. Compared with linear RNAs, circRNAs have stronger stability owing to their characteristic covalently closed loops without 5′ head or 3′ end structures. In recent years, circRNAs have become a hotspot in RNA research. Widely present in eukaryotic cells, they exert specific biological functions by regulating miRNAs and proteins [2, 3] and are closely involved in the occurrence, development, and prognosis of a variety of malignant tumors. Owing to their structural stability, wide distribution, and tissue specificity, circRNAs have advantages over other ncRNAs as molecular diagnostic markers and drug therapy targets. The expression of circRNAs in tumor tissues has been shown to differ significantly from that in normal tissues [4], indicating that circRNAs could serve as new tumor biomarkers for guiding clinical diagnosis. Known circRNAs can affect the biogenesis of cancers in diverse ways [4], such as acting as molecular sponges for miRNAs or competing endogenous RNAs (ceRNAs) to regulate the expression of target genes; they can also combine with RNA-binding proteins (RBPs) to regulate the expression of specific proteins, participate in protein translation and other biological functions, and indirectly participate in the occurrence and development of malignant tumors.

Large numbers of circRNAs stably exist in exosomes, which are involved in the composition and regulation of the microenvironment of malignant tumors. The circRNAs are delivered to normal cells via exosomes and have a significant impact on the progression and metastasis of malignant tumors. Recent exosome-related studies have further promoted the clinical translation of circRNAs in oncology, owing to the close connections between circRNAs and exosomes. Here, we summarize the biological functions, roles in diagnosis and treatment, possible mechanisms, and potential applications of exosome-derived circRNAs in malignant tumors.

## Structure and biological function of exosome

Exosomes were first identified in sheep reticulocytes and named by Johnstone. Transmission electron microscopy images show that they have a double-layer phospholipid structure with a “spherical” or “cup-like” shape and obvious heterogeneity, and they can be enriched in a concentration gradient of 1.13–1.19 g/ml sucrose solution. Exosomes are biologically active extracellular vesicles that are rich in lipids including cholesterol, ceramide, and sphingomyelin. They contain a variety of functional proteins [e.g., CD9, TSG101, and heat shock protein (HSP70)], DNA, miRNA, lncRNA (long non-coding RNA), and other biologically active substances, which are protected from destruction by being encapsulated in the exosome. The continuous formation process of exosomes mainly involves endocytosis, fusion, and exocytosis, that is, the invagination of the membrane to form a dynamic subcellular structure of multivesicular bodies. These multivesicular bodies have two outcomes: (1) they fuse with lysosomes, and the luminal vesicles are degraded; (2) they fuse with cell membranes, and the tubular vesicles inside depress again to form granular vesicles by endogenous budding and are then released into the extracellular environment in the form of exosomes [5, 6].

Exosomes can be detected in almost all normal and tumor cells and are present in a wide variety of body fluids including saliva, blood, urine, ascites, and breast milk [7, 8]. Their main function is to mediate intercellular communication and material exchange of their contents under physiological and pathological conditions [6]. By transmitting genetic and molecular messages from tumor cells to other cells located at near or distant sites to alter their functional properties and phenotypes [9], exosomes are also important carriers of specific signals for tumor proliferation, metastasis, immune response, angiogenesis, and drug resistance [10–12]. Studies have shown that tumor cells secrete more exosomes whose contents are different than normal cells. Tumor-cell-derived exosomes can provide a suitable microenvironment for tumor development and regulate cell proliferation, angiogenesis, extracellular matrix degradation, metastasis, drug resistance, and the formation of a pre-metastatic microenvironment [13, 14]. Tumor-derived exosomes are mainly composed of ncRNAs, including circRNAs, lncRNAs, and miRNAs. They are considered to be extracellular organelles that play essential parts in remodeling the tumor microenvironment, and represent a new type of non-invasive diagnostic biomarker for tumors [8]. Exosomes have been proved to promote tumor angiogenesis and metastasis during tumorigenesis [15], contributing to the formation of pre-metastatic ecological niches and the establishment of a suitable microenvironment at distant metastatic sites through stimulating neovascularization, activating mesenchymal stromal cells, and remodeling the extracellular matrix [16, 17]. Tumor-derived exosomes can participate in tumor microenvironment remodeling by releasing cytokines and other bioactive components that act on endothelial cell surface receptors, leading to angiogenesis and promoting tumor metastasis [18].

Studies have shown the main mechanisms of exosomes function are as follows [19]: direct interaction between exosomes and surface receptors on target cells; cleavage of surface receptors on exosomes and subsequent direct interaction of receptor fragments with receptors on target cells; fusion of exosome membranes with the plasma membrane of target cells, with release of informational material carried by exosomes into the cytoplasm and internalization of the entire exosome through phagocytosis.

## Structure and biological functions of circRNAs

Both circRNAs and linear RNAs originate from precursor mRNAs; however, circRNAs are formed by reverse splicing, in contrast to the production of linear RNAs by classical splicing. CircRNAs originate from all regions of the genome, including intergenic, intron, antisense, and untranslated regions. According to their origin, circRNAs can be classified into three major groups: exonic circRNAs, exon–intron circRNAs, and intronic circRNAs. CircRNAs are more stable and less susceptible to degradation by nucleic acid exonucleases than linear RNAs because of their chemical structure of covalent closed loops [2].

CircRNAs were initially thought to be non-functional byproducts resulting from splicing errors. However, with the development of high-throughput sequencing technology and bioinformatics research, large numbers of circRNAs have been found in mammalian cells, and their structural stability, high conservation, and abundant expression have been demonstrated [20]. The main biological functions of circRNAs: (1) competitive binding with miRNAs to regulate target gene expression; (2) interactions with RBPs to regulate gene expression; (3) regulation of gene transcription or splicing; (4) translation of proteins; and (5) regulation of epigenetic inheritance. Research has shown that circRNAs are related to the pathogenesis of many human diseases and have key roles in tumorigenesis, development, and metastasis; thus, they can be used as effective biomarkers for early diagnosis and prognostic prediction in tumor patients, as well as being ideal targets for early treatment [21].

## Biological functions of exosomal circRNAs

Exosomes are involved in the formation of the tumor microenvironment before metastasis. By mediating immunosuppression and immunosurveillance, exosomes are associated with tumor recurrence and tumor cell survival [22] Li et al. [3] found that large numbers of circRNAs were stably present in exosomes derived from various carcinoma cell lines [including laryngeal cancer, oral squamous cell carcinoma (OSCC), thyroid cancer, liver cancer, colon cancer, lung cancer, stomach cancer, and breast cancer] and could be detected in patients’ saliva, serum, urine, and tumor tissues. Various studies have reported that exosomal circRNAs may be related to the occurrence and development of malignant tumors and may regulate tumor cell proliferation, invasion, metastasis, chemotherapy drug resistance, and radiosensitivity.

### Regulation of tumor cells proliferation

Dysregulation of proliferation is an important factor in tumor transformation; therefore, the mechanisms of cell cycle regulation have been increasingly brought into focus. Recently, the role of exosomal circRNAs in regulating tumor cell proliferation in various tumors has been gradually elucidated. Exosome-derived circRASSF2 was found to promote the progression of laryngeal squamous cell carcinoma; circRASSF2 expression was significantly higher in tumor tissues than that in control tissues, and downregulation of exosomal circRASSF2 significantly inhibited cell proliferation via the miRNA-302b-3p/IGF-1R axis [23]. In hepatocellular carcinoma, arsenite was shown to induce malignant transformation of human liver epithelial cells (L-02) and cause overexpression of circ_100284, which could be transferred from transformed L-02 cells to normal cells via exosomes. Exosomal circ_100284 accelerated the cell cycle and promoted cell proliferation in normal cells by interacting with miRNA-217. This shows that exosomal circRNAs act as messengers in intercellular communication and suggests a mechanism of arsenite-induced carcinogenesis [24]. Exosomal circDB is upregulated in hepatocellular carcinoma (HCC) patients with high body fat ratio and is transferred to adjacent normal cells with the release of exosomes from malignant transformed cells. It can regulate deubiquitination in HCC by suppressing miRNA-34a and activating the USP7/cyclin A2 signaling pathway, thereby promoting abnormal hepatocyte proliferation and reducing DNA damage [25]. CircIFT80 can promote abnormal proliferation of colorectal cancer (CRC) cells through exosomes, promote DNA synthesis and inhibit apoptosis via the miRNA-1236-3p/HOXB7 axis [26]. CircFMN2 is highly expressed in serum exosomes of CRC patients and its expression levels is negatively correlated with miRNA-1182 levels. CircFMN2 binding to miRNA-1182 can significantly promote CRC cell proliferation, suggesting that exosomal circFMN2 plays a significant part in promoting tumor growth in CRC [27].

Yu et al. showed that the exosomal circNEK9 accelerated the proliferation, migration, and invasion of gastric cancer (GC) cells by targeting the miR-409-3p/MAP7 axis [28]. CircNRIP1 was significantly upregulated in GC tissues and acted as a miR-149-5p sponge to promote the proliferation, invasion, and metastasis of GC cells; inhibiting circNRIP1 could block the malignant behavior of GC cells via the AKT1/mTOR signaling pathway [29]. Circ_0009910 expression is upregulated in bone marrow, cells and exosomes of acute myeloid leukemia (AML). Inhibiting circ_0009910 can inhibit AML cell proliferation, block the cell cycle, and promote apoptosis. Circ_0009910 regulates AML cell behavior through exosome secretion and the circ_0009910-miR5195-3p/GRB10 ceRNA axis; therefore, circ_0009910 may be an effective target for the treatment of AML [30]. Li et al. [31] reported that circ_0044516 has a critical role in tumor cell survival and metastasis in prostate cancer. Circ_0044516 has been shown to be significantly upregulated in exosomes and cell lines of prostate cancer patients, and its downregulation inhibits the proliferation and metastasis of prostate cancer cells. Exosomal-derived circ_0044516 may promote prostate cancer cell proliferation by acting as a miRNA-29a-3p sponge. Ma et al. [32] showed that highly metastatic ovarian cancer EOC cells could transfer their characteristics to cells with low metastatic potential via exosomal circRNA051239, thereby enhancing the proliferation, migration, and invasion capabilities of recipient cells; this indicates a possible mechanism for ovarian cancer treatment.

### Regulation of tumors invasion and metastasis

Exosomes can mediate molecular communication and transfer of materials between the primary tumor site and distant metastatic sites, regulate a range of cellular activities including epithelial–mesenchymal transition (EMT), and have crucial roles in invasion and metastasis of tumor cells [33]. Exosomal circ_100338 has been shown [34] to be involved in the regulation of angiogenesis and metastasis in HCC and is closely associated with the progression of HCC. Transfer of exosomal circRNA-100338 to recipient human umbilical vein endothelial cells (HUVECs) can enhance the metastatic ability of HCC cells, and HUVECs affect pro-angiogenic activity by regulating angiogenesis. HCC cells with high metastatic potential can confer this potential on low-metastatic-potential and non-metastatic cells via exosomes, thereby increasing the invasive and metastatic capacity of these cells. Wang et al. [35] found that exosomal circPTGR1 contributes to the metastasis of HCC; high expression of circPTGR1 was closely associated with clinical stage and prognosis, and knocking out the expression of exosomal circPTGR1 could significantly inhibit tumor invasion and metastasis in low-metastatic-potential or non-metastatic cell lines. Exosomes from circPTGR1-enriched highly metastatic HCC cells may affect low-metastatic-potential HCC cells by downregulating miR449a-MET interactions in recipient cells, thereby disrupting the homeostasis of the tumor microenvironment and promoting HCC progression.

Given that circPTGR1 is highly abundant and aberrantly expressed in malignant cells and cells from patients with metastasis, it may serve as a prognostic biomarker and therapeutic target for HCC. Circ-0004277 promotes the malignant phenotype of HCC cells by inhibiting zonula occludens 1 (ZO-1) and regulating EMT progression, and stimulates peripheral cells EMT through intercellular communication, further promoting the invasion of HCC to surrounding normal tissues [36]. HCC cells that overexpress circCdr1as can promote the proliferation and metastatic ability of surrounding normal cells through exosome delivery [36]. Wang et al. [37] showed that exosomes from cholangiocarcinoma cells enhanced the expression of circ-0000284 and stimulated the proliferation and migration of surrounding normal cells. Circ-0000284 can either act as a ceRNA to promote the progression of cholangiocarcinoma, or it can be transferred directly from cholangiocarcinoma cells to surrounding normal cells via exosomes, thereby regulating the biological functions of the surrounding normal cells.

Exosomal circRNAs have significant roles in the progression and metastasis of pancreatic cancer (PC). Exosomal circIARS is widely expressed in PC tissues, where its levels are positively correlated with liver metastasis, vascular invasion, and TNM stage (liver metastasis: P = 0.011; vascular invasion: P = 0.020; TNM: P = 0.023). Circ-IRAS enters microvascular endothelial HUVECs via exosomes from PC cells, downregulates miRNA-122 and ZO-1, upregulates RhoA and RhoA-GTP, increases F-actin expression and focal adhesion, increases the permeability of endothelial monolayer, and promotes tumor invasion and metastasis [38]. Li et al. [39] showed that exosomal circPED8A was highly expressed in PC tissues and was associated with lymphatic invasion, TNM stage, and low survival rate. CircPDE8A can promote tumor cell growth through upregulating MET. CircPDE8A secreted by tumor cells can be released into the circulation via exosomes, regulate MACC1 as a miRNA-338 sponge, and promote invasion and metastasis through the MET/mitogen-activated protein kinase 1(MAPK1) or protein kinase B pathway.

Exosomal circRNAs can promote GC invasion and metastasis. CircNRIP1 can be transmitted between GC cells via exosomes and affect the Akt1/mTOR signaling pathway as a miRNA-149-5p sponge, thus promoting GC cell metastasis [29]. CircRanGAP1 is significantly upregulated in both cancer tissue and plasma exosomes of GC patients and enhances metastasis and invasion of GC cells via the miRNA-8773p/VEGFA axis [40]. Chen et al. studied exosomes in serum and urine of bladder urothelial carcinoma patients and found that circPRMT5 was significantly upregulated in both sample types [41]; circPRMT5 could act as a miRNA-30c sponge and promote EMT through the circPRMT5/miRNA30c/SNAIL1/E-calcine mucin 1 pathway. High expression of circPRMT5 in serum and urine exosomes was positively correlated with lymph node metastasis and advanced tumor progression (P < 0.05). Li et al. [42] showed that FLI1 exon circRNA is a novel oncogenic driver that promotes small cell lung cancer metastasis through the miR584/ROCK1 pathway, and proposed serum exosomal FECR1 as a promising biomarker that could be used to follow the disease progression of small-cell lung cancer. In ovarian cancer, exosomes transfer circPUM1 and circWHSC1 to peritoneal mesothelial cells, thereby promoting tumor metastasis [43, 44]. Studies related to CRC [26, 45] have also identified a role of exosomal circRNAs in promoting tumor invasion and metastasis.

### Modulation of chemotherapy resistance and radiosensitivity

Radiotherapy is an important tool for tumor treatment and has a particularly crucial role in the treatment of advanced malignant tumors. However, drug resistance and decreased sensitivity to radiotherapy inevitably occur, leading to tumor recurrence and metastasis [46, 47]; these are the main reasons for failure of tumor treatment and seriously affect treatment effects and prognosis. Therefore, there is an urgent need to overcome tumor drug resistance and enhance sensitivity to radiotherapy. Studies have shown [48] that exosomes can transmit drug resistance between GC cells. The essential role of circRNAs as the content of chemotherapy resistance transmission is gradually being revealed; for instance, exosomes derived from tumor-associated macrophages were found to transfer miR-21, leading to cisplatin (DDP) resistance in GC. The exosomal circ_0032821 promotes oxaliplatin (OXA) resistance in GC cells via the miR-515-5p/SOX9 axis, suggesting that circ_0032821 is expected to be an effective therapeutic target for GC [49].

Exosomal circRNAs have a vital role in regulating chemotherapeutic drug resistance and radiosensitivity of CRC. Wang et al. [47] found that exosomal circ_0067835 inhibited CRC progression and enhanced the radiosensitivity of CRC cells via the miR-296-5p/IGF1R axis. When circ_0000338 was selectively transferred to a co-cultured parental HCT116 cell line (HCT116-P) by exosomes secreted from an OXA-resistant HCT116 cell line (HCT116-R), the viability of HCT116-P cells during drug treatment significantly increased compared with control cells [50]. Malignant tumors usually rely on aerobic glycolysis to produce adenosine triphosphate for rapid growth and resistance to chemotherapeutic agents, and M2-PK has a key role in catalyzing glycolysis. Study has shown [51] that M2-PK expression is heterogeneous in CRC cells, being higher in OXA-resistant cells and lower in OXA-sensitive cells. miRNA-122 is positively correlated with chemoresistance; exosomes from OXA-resistant cells deliver ciRS-122 to sensitive cells to promote glycolysis and drug resistance by adsorbing miRNA-122 and upregulating M2-PK; this intercellular signaling provides a theoretical basis for the clinical applications of exosomes in drug-resistant CRC.

The exosomal circZNF91 can act as a transporter to mediate signaling between hypoxic and normoxic tumor cells and promote gemcitabine resistance in PC; therefore, it may be an effective therapeutic target [52]. Xu et al. [53] showed that exosomal hsa_circ_0014235 promotes DDP resistance and development of non-small-cell lung cancer (NSCLC) by mediating the miR-520a-5p/CDK4 pathway. Hsa_circ_0002130 has a promising role as a therapeutic target in osimertinib-resistant NSCLC [54]. Exosome-mediated circ_UBE2D2 has been shown to enhance tamoxifen resistance in breast cancer by binding to miR-200a-3p; this finding provides new insights that could be used to improve the efficacy of tamoxifen in breast cancer patients [55].

circCdr1as is involved in the formation of DDP resistance in ovarian cancer; functioning as a miRNA sponge to downregulate miRNA-1270 and upregulate suppressor of cancer cell invasion (SCAI), the target gene of miRNA-1270, it can enhance the sensitivity of ovarian cancer cells to DDP. Serum exosomal circCdr1as was found to be abnormally downregulated in a DDP-resistant group compared with the DDP-sensitive group and was significantly negatively correlated with expression levels of miRNA-1270 (r =  − 0.679, P = 0.000) [56]. Serum exosome hsa_circ_103801 could serve as a valid prognostic biomarker for osteosarcoma (OS) and may be a potential target for overcoming OS chemoresistance [57].

## Role of exosomal circRNAs in diagnosis and prognosis

A certain amount of progress has been made with respect to the treatment of malignant tumors, for example, in early diagnosis, surgery, and radiotherapy; however, the early symptoms of most tumors are not typical and final diagnosis is difficult as it requires specialized clinicians to obtain samples for processing and to perform complex histopathological biopsies. There is still a lack of rapid, accurate, and non-invasive early diagnostic biomarkers for use in clinical practice. Liquid biopsy based on exosomal circRNAs is a novel method, taking advantage of the fact that circRNAs are enriched in exosomes and have good stability, and that the expression profile of exosomal circRNAs in patients with malignant tumors is different from that in the healthy population [22]. Liquid biopsy can be easily performed using samples of saliva, serum, urine, and other body fluids, avoiding the harm caused to patients by surgery or puncture. As well as its advantages in terms of safety, this method is easier to perform [58]; it also enables monitoring of the whole process of malignant tumor diagnosis and prognosis evaluation, with strong real-time and timeliness. Thus, exosomal circRNAs have important diagnostic value in a variety of malignant tumors and have great potential as molecular markers for non-invasive early diagnosis and prognostic assessment, thereby providing a new direction for exploration of powerful diagnostic markers for different tumors. Although exosomal circRNAs have great potential to become novel tumor markers, the current understanding of exosomal circRNAs is still in its infancy. Further studies are needed to verify whether the proposed circRNAs have clinical value as tumor markers.

Studies have shown that exosomes are rich in circRNAs with potential biological functions, and human serum exosomes have been proven to contain more than 1000 types of circRNA. This phenomenon is caused by the entry of circRNAs from tumors into the bloodstream [59]. CircRNAs are stably enriched in malignant exosomes, and the transport of circRNAs and other types of molecules by exosomes protects them from degradation and dilution in the cellular extracellular space, resulting in long-distance distribution of circRNAs via the bloodstream or tissue fluid [60]. To detect whether exosomal circRNAs enter the blood circulation and can be quantified for malignancy diagnosis, Xu et al. [61] analyzed the expression of exosomal circRNAs in serum of endometrial cancer patients and healthy individuals by an RNA sequencing technique. They found that the number of circRNAs upregulated in exosomes of endometrial cancer patients was higher than the number of downregulated circRNAs, indicating that human malignant circRNAs can enter the blood and be easily quantified in serum, and that exosomal circRNAs can affect target cells. These findings may help to identify new mechanisms of malignant tumor development. Li et al. [3] constructed a mouse tumor model and detected human circRNA-CDYL in serum exosomes; the amount of circRNA-CDYL was closely related to tumor development, further confirming that circRNAs can be used as early diagnostic biomarkers of malignant tumors.

Han et al. [62] showed that plasma circ_0019201, circ_0011773, and circ_0122790 are potential biomarkers for predicting LSCC. Circ_0019201, circ_0011773, and circ_0122790 were found to be consistent with the training set. The receiver operating characteristic (ROC) curves also showed high diagnostic power, as did the area under the ROC curve (AUC) for both single and combined circRNAs. Circ_0019201, circ_0011773, circ_0122790, and their combinations had AUCs of 0.933, 0.908, 0.965, and 0.990 in the training set and 0.766, 0.864, 0.908, and 0.951 in the validation set.

CircRNAs have a significant role in the development of esophageal squamous carcinoma (ESCC). The expression of serum exosomal hsa_circ_0026611 was found to be significantly upregulated in ESCC with lymph node metastasis [63]; exosomal hsa-circ-0048117 may have key roles in remodeling the ESCC microenvironment and regulating the progression of ESCC [64]; and hsa_circ_0001946 shows potential as a good prognostic biomarker [65]. Thus, circRNAs are expected to emerge as new markers for the diagnosis and prognostic assessment of ESCC patients.

In CRC studies, exosomal circRNA levels have been shown to be significantly upregulated in K-ras wild-type CRC DKs-8 cells, suggesting that exosomal circRNA could be used as a biomarker for CRC [66]. Serum exosomal circ_0004771 was found to be significantly upregulated in CRC patients with different TNM stages compared with the control group (P = 0.017), and its AUC for the diagnosis of CRC was 0.88, with a sensitivity of 80.91% and specificity of 82.86%; serum exosomal circ_0004771 in patients with stage I-II CRC was significantly higher than that in normal controls (P < 0.001), and the AUC for the diagnosis of stage I-II CRC was 0.86, with a sensitivity of 81.43% and specificity of 80.00% [67]. Barbagallo et al. [68] found that serum exosomal circHIPK3 was significantly upregulated in patients with CRC, and the AUC for the diagnosis of CRC was 0.771, with a sensitivity of 71% and a specificity of 80%.

Xie et al. [69] showed that exosomal circSHKBP1 regulates the miR-582-3p/HUR/VEGF pathway, inhibits HSP90 degradation, and promotes GC progression. CircSHKBP1 is thus a promising circulation biomarker for GC diagnosis and prognosis. Circ-RanGAP1 is significantly upregulated in both GC tissues and plasma exosomes of GC patients and is closely associated with advanced TNM stage, lymph node metastasis, and low survival; therefore, it could be used as a prognostic biomarker and therapeutic target for GC [39]. Shao et al. [70] found that plasma exosomal circ_0065149 levels in patients with early GC (EGC) were significantly lower than those in healthy controls (P < 0.001), and the AUC for screening for EGC was 0.64, with higher sensitivity (48.7%) and specificity (90.2%) than traditional tumor markers (CEA, CA199, and CA125).

Studies in HCC have confirmed [71] seven upregulated and five downregulated circRNAs, hsa_circ_0004001, hsa_circ_0004123, and hsa_circ_0075792, which among the upregulated circRNAs, the combination of which can be used as a valuable diagnostic biomarker for HCC. Exosome-derived circ_0051443, which is downregulated in the plasma of HCC patients, inhibited the progression of HCC and had an AUC of 0.8089 for the diagnosis of HCC [72]. Pancreatic ductal adenocarcinoma (PDAC) is among the most aggressive and lethal malignancies, with a 5-year survival rate of only 5% owing to the high risk of metastasis and recurrence. Microarray analysis [39] showed that circ-PDE8A is a highly expressed circRNA in PDAC and may play a critical part in PDAC invasion; thus, exosomal circ-PDE8A may be an ideal biomarker for early diagnosis or assessment of progression of PDAC. Tumor-derived exosomal circRNA_102481 may promote resistance to epidermal growth factor receptor tyrosine kinase inhibitors (EGFR-TKIs) in NSCLC via the miRNA-30a-5p/ROR1 axis. Exosomal circRNA_102481 therefore has potential as a novel diagnostic biomarker and therapeutic target in EGFR-TKI-resistant NSCLC [73]. Exosomal circFARSA plays a crucial part in the interaction between macrophages and NSCLC cells through the PTEN/PI3K/AKT signaling pathway and is a promising diagnostic/prognostic biomarker for NSCLC [74].

Li et al. [42] found that expression of serum exosomal FLI1 exonic circRNA 1 (FECR1) was abnormally upregulated in patients with small-cell lung cancer; moreover, higher levels of exosomal FECR1 expression were observed in patients with distant metastases and serum FECR1 levels were closely related to patients’ response to chemotherapy, indicating that FECR1 could be used as a biomarker to assess the progression of small-cell lung cancer. Wang et al. [75] found that serum exosomal circ_002178 was significantly upregulated in lung adenocarcinoma (LUAD) patients and enhanced expression of programmed cell death ligand 1 (PDL1) by adsorbing miRNA-34 and miRNA-28-5p; circ_002178 showed potential as a non-invasive diagnostic marker for LUAD with an AUC of 0.9967.

Chen et al. [76] comprehensively analyzed the expression profiles of plasma exosomal circRNAs in LUAD groups and healthy controls and found that the expression levels of circ_0001492 and circ_0001346 were upregulated in early-stage LUAD but almost undetectable in plasma exosomes of healthy controls, suggesting that circ_0001492 and circ_0001346 could be used as novel early diagnostic markers for LUAD. Lin et al. [77] extended current knowledge regarding the differential expression of circRNAs in plasma exosomes of advanced LUAD, offering prospects for further research on the development and use of biomarkers for diagnosis and prognosis of LUAD. Circ_0102537 was proved to be an effective diagnostic biomarker for advanced LUAD. Chen et al. reported for the first time the altered expression of exosomal circRNAs in plasma samples from LUAD patients, supporting the necessity to explore its usage as a biomarker and its pathological function in lung cancer [76]. Xu et al. [78] found that circSETDB1 promoted tumor formation through the miR-7/Sp1 pathway. Tumor-secreted circSETDB1 can be released into the circulation via exosomal transport under hypoxic stress, and plasma exosomal circSETDB1 is associated with lymphatic metastasis and T-stage in LUAD patients; thus, it may be an important biological factor in the development of LUAD and is expected to be an ideal biomarker for early diagnosis.

Bioinformatics analysis and real-time fluorescence quantitative polymerase chain reaction technology have been used to show that circ_00005795 and circ_0088088 can compete for endogenous RNAs, and both of these circRNAs were shown to have important diagnostic value in breast carcinoma by comparing the expression of serum exosomal circRNAs in breast carcinoma patients and healthy controls. These results provide a theoretical basis for the occurrence, progression, and metastasis of breast carcinoma [79, 80]. Circ-0001068 is introduced into T cells and induces PD1 expression via exosomes as a ceRNA for miR-28-5p; it represents a novel ovarian cancer biomarker and inducer of PD1 expression in T cells [81]. Targeting exosomal circ-CYP24A1 was found to inhibit the progression of cutaneous squamous cell carcinoma by attenuating the malignant behavior of the tumor [82]. It is thus a promising therapeutic target and non-invasive diagnostic biomarker for cutaneous squamous cell carcinoma.

Luo et al. [83] showed that circulating exosomal circMYC has great potential as a diagnostic and prognostic biomarker for multiple myeloma. Chemotherapy resistance limits the durability of therapeutic effects and is a major constraint to clinical treatment of oncology. Timely identification of potential drug-resistant patients, monitoring of resistance to chemotherapeutic agents, and finding alternative treatment options are extremely important strategies, enabling treatment to be adjusted and prognosis improved. Exosome-mediated circNFIX enhances temozolomide (TMZ) resistance in glioma by sponging of miR-132 and shows potential as a prognostic biomarker to improve the clinical benefit of TMZ treatment in glioma patients [84]. Exosome-mediated circ-HIPK3 enhances TMZ resistance via modulating the miR-421/ZIC5 axis and promotes tumorigenesis in vitro and in vivo; its expression in serum exosomes can be used as a biomarker for diagnosis of TMZ-resistant glioma [85].

The roles of exosomal circRNAs in diagnosis and prognostic assessment of malignant tumors are shown in Table 1.

**Table 1:** Role of exosomal circRNAs in diagnosis and prognostic assessment of malignant tumors

Tumor type	circRNA(s)	Expression level	Role(s)	References
HCC	circRNA-CDYL	Up	Diagnosis	[3]
LSCC	circ_0019201	Up	Diagnosis	[62]
	circ_0011773	Up	Diagnosis	[62]
	circ_0122790	Up	Diagnosis	[62]
ESCC	circ_0026611	Up	Prognosis	[63]
	circ-0048117	Up	Prognosis	[64]
	circ_0001946	Up	Prognosis	[65]
CRC	circ_0004771	Up	Diagnosis	[67]
	circHIPK3	Up	Diagnosis	[68]
GC	circSHKBP1	Up	Diagnosis and prognosis	[69]
	circ_0065149	Down	Diagnosis	[70]
HCC	circ_0004001	Up	Diagnosis	[71]
	circ_0004123	Up	Diagnosis	[71]
	circ_0075792	Up	Diagnosis	[71]
	circ_0051443	Down	Diagnosis and prognosis	[72]
PDAC	circ-PDE8A	Up	Diagnosis and prognosis	[39]
NSCLC	circRNA_102481	Up	Diagnosis	[73]
	circFARSA	Up	Diagnosis and prognosis	[74]
SCLC	FECR1	Up	Diagnosis and prognosis	[42]
LUAD	circ_002178	Up	Diagnosis	[75]
	circ_0001492	Up	Diagnosis	[76]
	circ_0001346	Up	Diagnosis	[76]
	Circ_0102537	Up	Diagnosis and prognosis	[77]
	circSETDB1	Up	Diagnosis and prognosis	[78]
Breast carcinoma	circ_00005795	Up	Diagnosis and prognosis	[79]
	circ_0088088	Up	Diagnosis and prognosis	[80]
Ovarian cancer	Circ-0001068	Up	Diagnosis	[81]
cSCC	circ-CYP24A1	Up	Diagnosis	[82]
MM	circMYC	Up	Diagnosis and prognosis	[83]
Glioma	circNFIX	Up	Prognosis	[84]
	circ-HIPK3	Up	Diagnosis	[85]

*HCC* hepatocellular carcinoma; *LSCC* laryngeal squamous cell carcinoma; *ESCC *esophageal squamous cell carcinoma; *CRC* colorectal cancer; *HCC* hepatocellular carcinoma; *PDAC* pancreatic ductal adenocarcinoma; *NSCLC* non-small cell lung cancer; *LUAD* lung adenocarcinoma; *SCLC* small-cell lung cancer; *GC* gastric cancer; *cSCC* cutaneous squamous cell carcinoma; *MM* multiple myeloma

## Role of exosomal circRNAs in treatment

The therapeutic delivery of exosomal circRNAs and related genes and proteins as targets can inhibit the proliferation, invasion, and metastasis of malignant tumor cells. This approach can also provide new ideas for therapy of tumors including OSCC [86], CRC [87, 88], GC [89], PC [90], cervical cancer [91, 92], gallbladder cancer [93], breast cancer [94], and OS [95, 96]. Exosomes can transport RNA or deliver therapeutic drugs to cancer cells and act as drug carriers for targeted therapy owing to their unique advantages, such as their nano nature, double lipid membrane, ability to serve as multiple carriers, good histocompatibility, high bioavailability, low cytotoxicity and immunogenicity, long life span, and high cargo capacity [97–99]. Exosomes can further target tumor cells through surface receptors, reducing side effects on healthy tissue. The stability of circRNAs and their function as miRNA molecular sponges suggest that circRNAs could be used as drug therapy targets. A variety of circRNAs have been proved to promote cancer progression. Moreover, whereas drugs such as specific small interfering RNAs (siRNAs) targeting specific circRNAs are inherently unstable and easily degraded, their encapsulation in exosomes can maintain the stability of siRNAs and reduce the expression of circRNAs in cancer cells. Exosomal circRNAs can also promote the expression of tumor suppressor genes through sponge-like uptake of miRNAs and indirectly inhibit the damage caused by circRNAs. These facts provide potential new approaches to tumor therapy using exosomal circRNAs. Tumor suppressor circRNAs are downregulated in tumors, and overexpression of circRNAs in tumor cells through exosomes may inhibit tumor progression; cancer-promoting circRNAs are upregulated in tumors, and siRNAs targeting circRNAs delivered by exosomes may downregulate the expression of cancer-promoting circRNAs and thus inhibit tumor progression. Given the important biological functions of exosomal circRNAs in tumor chemoresistance, the therapeutic use of RNAs targeting exosomal circRNAs has the potential to reverse tumor chemoresistance. Exosomal circRNAs have promising clinical applications as targets for tumor therapy. However, circRNAs are still in the research stage, and the clinical applications of exosomal circRNAs require deeper exploration.

Upregulation of circ_0000199 in circulating exosomes of OSCC patients is positively correlated with poor survival; thus, circulating exosomal circ_0000199 could be used as a diagnostic biomarker and potential therapeutic target in OSCC patients [100]. By combining the in vitro and in vivo studies, exosomal circGDI2 can regulate the malignant behavior of OSCC cells by targeting the miR-424-5p/SCAI axis and is expected to serve as an exosome-based biomarker and therapeutic target for OSCC [101]. The circulating exosomes were obtained from 210 patients with nasopharyngeal carcinoma (NPC); the result suggested that circMYC is an oncogene in NPC cells that enhances the resistance of NPC cells to radiotherapy; circMYC in circulating exosomes could be a potential therapeutic target for NPC [102]. CircFNDC3B has been shown to regulate progression of papillary thyroid cancer (PTC) progression in tissues and cell lines through the miR-1178/TLR4 pathway [103]; it may thus be an effective therapeutic target for the treatment of PTC.

A series of in vitro and in vivo assays were applied to elucidate that circLONP2 acts as a key metastasis initiator in CRC progression by regulating intracellular maturation and intercellular transfer of miR-17, promoting the ability to initiate metastasis at the primary site and the formation of metastatic foci in other tissues. Therefore, it could serve as an effective prognostic predictor and as a new target for anti-metastatic therapy in CRC treatment [104]. Transfecting specific siRNAs in CRC cells was shown to inhibit the expression of circRNA-ACAP2 and circCCDC66 and significantly reduced cell proliferation, invasion, and metastasis in the treatment group compared with the control group [87, 88]. Exosomal circ_IFT80 promotes tumorigenesis and reduces radiosensitivity by regulating the miR-296-5p/MSI1 axis in vitro and in vivo, potentially indicating a new strategy for the treatment of CRC [105].

Bone marrow mesenchymal stem cell (BM-MSCs) exosomal circ_0030167 promotes progression and acquisition of stem cell properties in PC cell lines through the miR-338-5p/wif1/Wnt 8/β-catenin axis, offering new prospects for PC treatment [106]. Ye et al. [107] showed that hsa_circ_0000069 gene downregulation could inhibit the development of pancreatic carcinogenesis and the malignant transformation of HPDE cells, indicating that it may be an effective therapeutic target for PC therapy. The interplay network of hsa_circ_0002130–hsa_miR_4482-3p–NBN suggests the possible existence of sponge-attracting miRNAs and target mRNAs, and indicates a role for circRNAs in exploring the molecular mechanisms of PC cell reaggregation after radiotherapy, as well as their potential function as therapeutic targets [108].

Exosomal circ_0061395 promotes the growth of HCC cells by competitively binding to miR-877-5p and increasing the expression of PIK3R3, and could therefore be an effective target for HCC therapy [109]. Exosomal circUHRF1, which is mainly secreted by HCC cells, can promote immunosuppression by inducing natural killer cell dysfunction, which may lead to resistance to anti-PD1 immunotherapy and provides a potential therapeutic strategy for HCC patients [110]. Data have shown that circRNAs are abundant and stable in exosomes and are not only novel participants in the pathogenesis of cholangiocarcinoma but also good noninvasive diagnostic biomarkers in vitro and in vivo [111].

In renal cell carcinoma, circ_400068 has an important regulatory role, and the circ_400068/miR-210-5p/SOCS1 axis may be a candidate therapeutic target for renal cell carcinoma treatment in vitro and in vivo [112]. Li et al. [113] showed that circPRRC2A can competitively bind miR-514a-5p and miR-6776-5p and reduce the degradation of the tissue-specific oncogene TRPM3, which in turn promotes tumor growth and invasion. High expression of circPRRC2A is positively correlated with clinical stage and decreased survival in patients with advanced renal cell carcinoma and is expected to become a therapeutic target for renal cell carcinoma. Tumor-derived exosomal circPSMA1 promotes development, invasion, and metastasis of triple-negative breast cancer (TNBC) via the miR-637/Akt1/β-catenin (cyclin D1) axis, providing a new perspective from which to explore new potential biomarkers and immunotherapeutic strategies for TNBC in vitro and in vivo [114]. CircHIF1A from the exosomes of hypoxic cancer-associated fibroblasts (CAFs) has an important role in maintaining the stem cell properties of breast cancer cells, and circHIF1A may serve as an effective target molecule for breast cancer therapy [115].

Han et al. [116] showed that exosomal circRNA_0001445 promotes glioma progression through the miRNA-127-5p/SNX5 signaling pathway in vitro, providing new insights that could be used to explore the molecular mechanisms of this process. CircRNA_0001445 is thus a promising therapeutic target for glioma. CircSMARCA5 acts as a potent pharmacological tumor suppressor in glioblastoma and functions by bundling RBP SRSF1 in vitro and in vivo [117]. Li et al. [118] found that the transfer of circ-0000190 encapsulated by extracellular vehicles (EVs) from normal cells to OS cells could reduce the proliferation, invasion, and migration ability of OS cells, suggesting that EVs encapsulating circ-0000190 could be constructed and used to affect intercellular communication between normal cells and OS cells, thereby inhibiting the progression of OS.

## Regulatory factors and mechanisms of exosomal circRNAs

The clarification of biological functions forms a basis for probing ideal tumor markers, and identifying the regulatory factors of exosomal circRNAs and the mechanisms by which they affect malignant tumor cell growth is the basis for deciding whether exosomal circRNAs can become novel diagnostic markers and therapeutic targets for malignant tumors. The causes and mechanisms of tumor growth are very complex and include regulation of tumor cells apoptosis and proliferation, invasion and metastasis, and modulation of tumor chemotherapy resistance and radiosensitivity.

### Regulatory factors of exosomal circRNAs

Several recent studies have shown that multiple regulatory factors can modulate the expression of exosomal circRNAs in malignancies, e.g., sevoflurane, arsenite, hypoxia, and matrine. Sevoflurane inhibits CRC progression by modulating the circ-HMGCS1/miR-34a-5p/SGPP1 axis when delivered by exosomes [119]. Hypoxia-derived exosomal circ-133 is transferred into normoxic CRC cells and promotes CRC cell migration through the miR-133a/GEF-H1/RhoA axis; this finding reveals a potential mechanism by which intra-tumor oxygen heterogeneity promotes tumor progression [120]. Gu et al. [121] showed that matrine inhibited the occurrence of CRC by blocking the release of exosomal circSLC7A6 from CAFs, providing a strong rationale for the use of matrine in the treatment of CRC.

Dai et al. [24] showed that long-term exposure to arsenite could induce malignant transformation and overexpression of circRNA_100284 in L-02 cells, and that circRNA_100284 could be transported from transformed L-02 cells to normal cells via exosomes. In normal cells, exosomal circRNA_100284 induced cell cycle acceleration and promoted proliferation by interacting with miR217, suggesting that arsenite exerts its oncogenic effects via a mechanism involving induction of circRNAs.

### Possible mechanism of exosome circRNAs

**Fig. 1:**
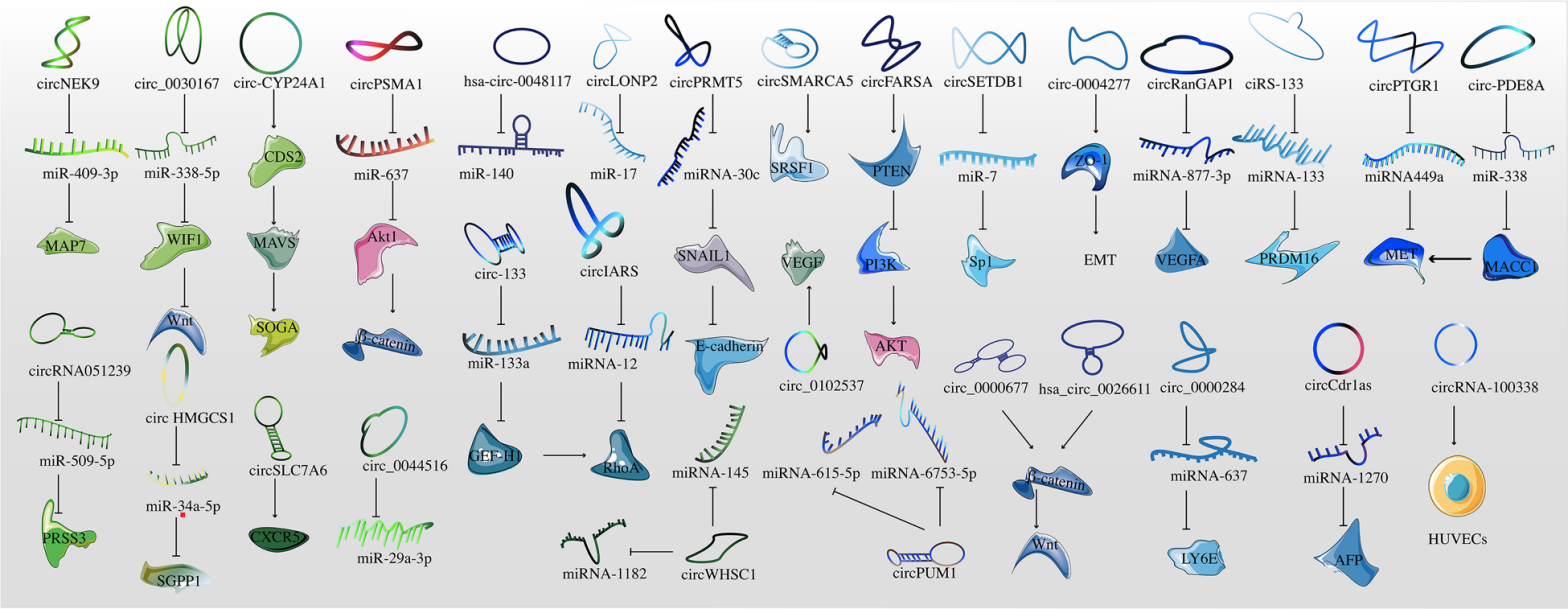
The target molecules of exosomal circRNAs in a variety of malignancies. It shows the roles of invasion and metastasis

**Fig. 2:**
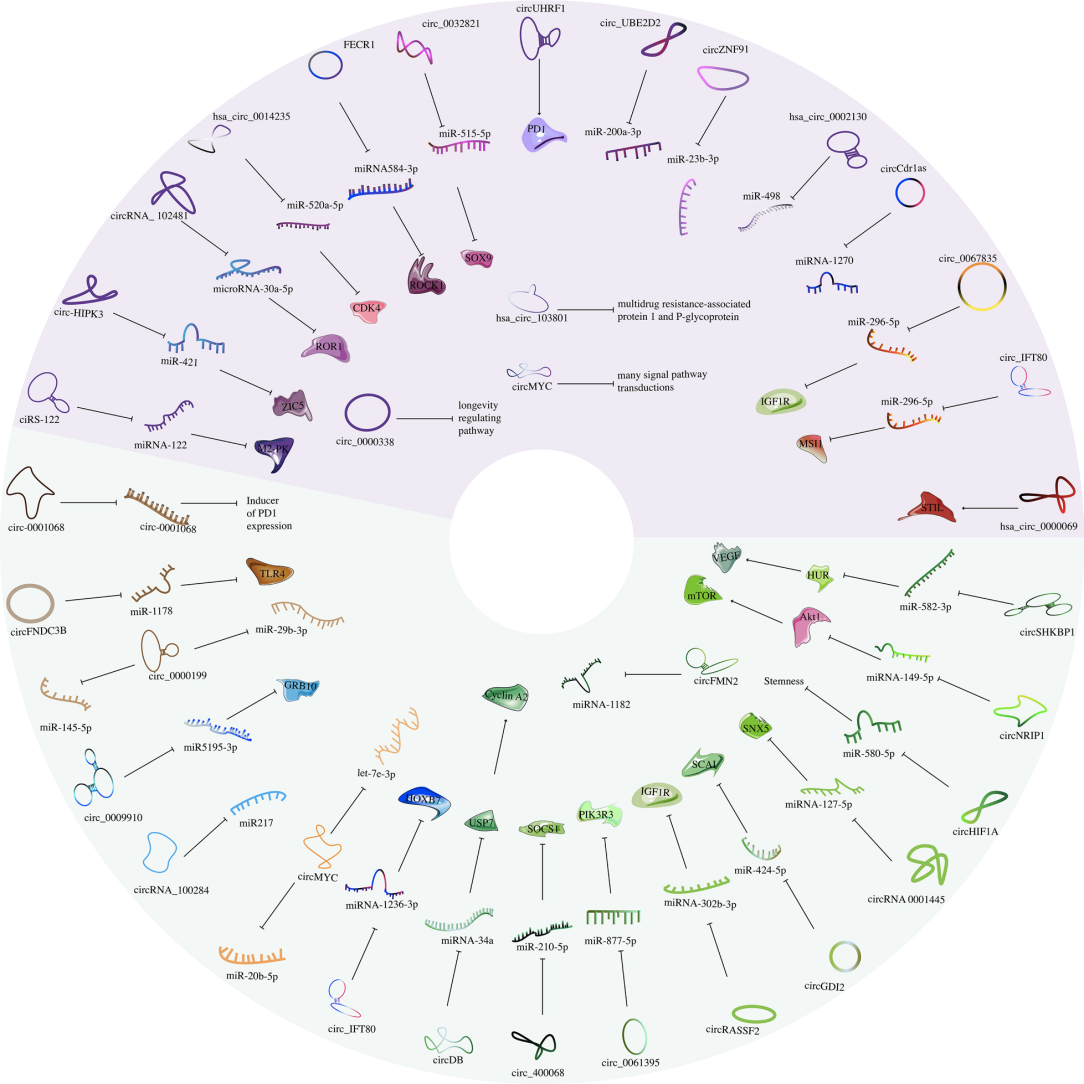
The target molecules of exosomal circRNAs in a variety of malignancies. The purple part shows the roles of chemotherapy resistance and radiosensitivity, the green part shows the roles of apoptosis and proliferation

CircRNAs have been confirmed to exist in exosomes, suggesting a new direction for the study of circRNAs. CircRNAs are mostly located in the cytoplasm, and the types of circRNAs in exosomes vary according to the type of cytoplasm, suggesting that the transfer of specific circRNAs to exosomes may be a positively regulated and selective process. Exosomal circRNAs can regulate tumor growth by regulating tumor cells apoptosis and proliferation, invasion and metastasis, and modulating tumor chemotherapy resistance and radiosensitivity. Exosomal circRNAs can target multiple oncogenes or proteins, e.g., miRNA-302b-3p/IGF-1R, miR-424-5p/SCAI, miR-1178/TLR4, and Wnt/β-catenin, to promote their expression and enable them to exert their growth-promoting effects. The target molecules of exosomal circRNAs in a variety of malignancies are shown in Table 2, Figs. 1 and 2.

**Table 2:** Target molecules of exosomal circRNAs and consequences of their targeting in malignant tumors

Tumor type	circRNA(s)	Target molecule(s)	Effect(s)	References
LSCC	circRASSF2	miRNA-302b-3p/IGF-1R	Proliferation	[23]
OSCC	circGDI2	miR-424-5p/SCAI	Progression	[101]
	circ_0000199	miR-145-5p, miR-29b-3p	Proliferation, apoptosis	[100]
NPC	circMYC	miR-20b-5p, let-7e-3p	Proliferation, radiosensitivity	[102]
PTC	circFNDC3B	miR-1178/TLR4	Proliferation, apoptosis	[103]
ESCC	hsa-circ-0048117	miR-140	Invasion, metastasis	[64]
	hsa_circ_0026611	Wnt/β-catenin	Lymph node metastasis	[63]
CRC	circSLC7A6	CXCR5	Proliferation, invasion, apoptosis	[121]
	circ-133	miR-133a/GEF-H1/RhoA	Metastasis	[120]
	circ-HMGCS1	miR-34a-5p/SGPP1	Proliferation, invasion, apoptosis	[119]
	circ_IFT80	miR-296-5p/MSI1	Tumorigenesis, radiosensitivity	[105]
	circLONP2	miR-17	Invasion, metastasis	[104]
	circ_0067835	miR-296-5p/IGF1R	Radiosensitivity	[47]
	circIFT80	miRNA-1236-3p/HOXB7	Proliferation	[26]
	circFMN2	miRNA-1182	Proliferation	[27]
	circ_0000338	longevity regulating pathway	Resistance	[50]
	ciRS-122	miRNA-122/M2-PK	Resistance	[51]
	circ_0000677	Wnt/β-catenin	Invasion, metastasis	[45]
HCC	circRNA_100284	miR217	Proliferation, cell cycle	[24]
	circUHRF1	PD1	Resistance	[110]
	circ_0061395	miR-877-5p/PIK3R3	Progression	[109]
	circ-0004277	ZO-1, EMT	Invasion	[36]
	circPTGR1	miR449a-MET	Metastasis	[35]
	circRNA-100,338	HUVECs	Metastasis	[34]
	circDB	miRNA-34a/USP7/Cyclin A2	Proliferation	[25]
	circPTGR1	miRNA449a/MET	Metastasis	[35]
	circ_100338	HUVECs	Metastasis	[34]
	circCdr1as	miRNA-1270/AFP	Invasion, metastasis	[122]
Cholangiocarcinoma	circ_0000284	miRNA-637/LY6E	Invasion, metastasis	[37]
GBM	circSMARCA5	RBP SRSF1	Metastasis	[117]
Glioma	circRNA 0001445	miRNA-127-5p/SNX5	Progression	[116]
	circ-HIPK3	miR-421/ZIC5	TMZ resistance	[85]
PC	hsa_circ_0000069	STIL	Tumorigenesis	[107]
	circ_0030167	miR-338-5p/wif1/Wnt 8/β-catenin	Proliferation, invasion, metastasis	[106]
	circ-PDE8A	miR-338/MACC1/MET	Invasion	[39]
	circZNF91	miR-23b-3p	GEM resistance	[52]
	circPED8A	miRNA-338/MACC1/MET	Invasion, metastasis	[52]
	circIARS	miRNA-12/RhoA	Invasion, metastasis	[38]
PDAC	circ-IRAS	endothelial monolayer permeability	Metastasis	[38]
TNBC	circPSMA1	miR-637/Akt1/β-catenin (cyclin D1)	Tumorigenesis, metastasis	[114]
Breast cancer	circHIF1A	miR-580-5p	Proliferation; stemness	[115]
	circ_UBE2D2	miR-200a-3p	Tamoxifen resistance	[55]
NSCLC	circFARSA	PTEN/PI3K/AKT	Metastasis	[83]
	circRNA_102481	microRNA-30a-5p/ROR1	EGFR-TKI resistance	[73]
	hsa_circ_0002130	miR-498	Osimertinib resistance	[73]
	hsa_circ_0014235	miR-520a-5p/CDK4	DDP chemoresistance	[53]
LUAD	circSETDB1	miR-7/Sp1	Invasion, EMT	[78]
	circ_0102537	VEGF	Invasion, metastasis	[77]
SCLC	FECR1	miRNA584-ROCK1	Resistance	[42]
Ovarian cancer	circ-0001068	miR-28-5p	Inducer of PD1 expression	[81]
	circRNA051239	miR-509-5p/PRSS3	Proliferation, metastasis	[32]
	circPUM1	miRNA-615-5p, miRNA-6753-5p	Invasion, metastasis	[43]
	circWHSC1	miRNA-1182, miRNA-145	Invasion, metastasis	[44]
	circCdr1as	miRNA-1270	Resistance	[56]
GC	circSHKBP1	miR-582-3p/HUR/VEGF	Progression	[69]
	circ_0032821	miR-515-5p/SOX9	OXA resistance	[49]
	circNEK9	miR-409-3p/MAP7	Proliferation, invasion, metastasis	[28]
	circNRIP1	miRNA-149-5p/AKT1/mTOR	Proliferation	[29]
	circRanGAP1	miRNA-877-3p/VEGFA	Invasion, metastasis	[40]
	ciRS-133	miRNA-133/PRDM16	Invasion, metastasis	[123]
Prostate cancer	circ_0044516	miR-29a-3p	Proliferation, metastasis	[31]
	circ_0044516	miRNA-29a-3p	Proliferation, metastasis	[31]
UCB	circPRMT5	miRNA-30c/SNAIL1/E-cadherin	Invasion, metastasis	[41]
RCC	circ_400068	miR-210-5p/SOCS1	Proliferation	[112]
cSCC	circ-CYP24A1	CDS2, MAVS, SOGA	Proliferation, invasion, metastasis	[82]
OS	hsa_circ_103801	multidrug resistance-associated protein 1 and P-glycoprotein	Cisplatin resistance	[57]
MM	circMYC	many signal pathway transductions	Recurrence, bortezomib resistance	[83]
AML	circ_0009910	miR5195-3p/GRB10	Proliferation, cell cycle, apoptosis	[30]

*OSCC* oral squamous cell carcinoma; *NPC* nasopharyngeal carcinoma; *PTC* papillary thyroid carcinoma; *GBM* glioblastoma multiforme; *GEM* gemcitabine; *PC* pancreatic cancer; *TNBC* triple-negative breast cancer; *UCB* urothelial carcinoma of the bladder; *RCC* renal cell carcinoma; *OS* osteosarcoma; *AML* acute myeloid leukemia

### Conclusions

Exosomal circRNAs that can regulate tumor cell proliferation, invasion, metastasis, and drug resistance represent a new hotspot in tumor research. CircRNAs have highly conserved characteristics and tissue-specific expression patterns; they can be enriched in exosomes and are easily detected in various body fluids. Therefore, exploring the association between exosomes and circRNAs will not only have a profound impact on our understanding of the functions and characteristics of exosomes but it will also provide new strategies to inhibit tumor progression and reverse drug resistance, as well helping to identify new potential tumor biomarkers and therapeutic targets. At present, only a few circRNAs have defined functions or clinical applications, and our understanding of exosomal circRNAs is still very limited; a large number of exosomal circRNAs and their mechanisms of actions have not been identified. The diagnostic value of exosomal circRNAs in different tumors still lacks experimental verification. The advantages of exosomal circRNAs over circulating free circRNAs has also not been fully validated. Therefore, exosomal circRNAs are expected to be a focus of future research, and their clinical application value needs to be supported by sufficient theoretical results.

## Data Availability

Not applicable.

## Abbreviations

circRNA: Circular RNA
miRNA: MicroRNA
ncRNAs: Non-coding RNAs
ceRNAs: Competing endogenous RNAs
RBPs: RNA-binding proteins
lncRNA: Long non-coding RNA
HSP: Heat shock protein
EMT: Epithelial-mesenchymal transition
HUVECs: Human umbilical vein endothelial cells
MAPK1: Mitogenactivated protein kinase 1
AUC: Area under curve
EGC: Early gastric cancer
FECR1: FLI1 exonic circular RNAs 1
PDL1: Programmed cell death ligand 1
BM-MSCs: Bonemarrow mesenchymal stem cells
EVs: Extracellular nanovesicles
CAFs: Carcinoma-associated fibroblasts
LSCC: Laryngeal squamous cell carcinoma
OSCC: Oral squamous cell carcinoma
NPC: Nasopharyngeal carcinoma
PTC: Papillary thyroid carcinoma
ESCC: Esophageal squamous cell carcinoma
CRC: Colorectal cancer
HCC: Hepatocellular carcinoma
GBM: Glioblastoma multiforme
GEM: Gemcitabine
PC: Pancreatic cancer
PDAC: Pancreatic ductal adenocarcinoma
TNBC: Triple-negative breast cancer
NSCLC: Non-small cell lung cancer
LUAD: Lung adenocarcinoma
SCLC: Small-cell lung cancer
GC: Gastric cancer
UCB: Urothelial carcinoma of the bladder
RCC: Renal cell carcinoma
cSCC: Cutaneous squamous cell carcinoma
OS: Osteosarcoma
MM: Multiple myeloma
AML: Acute myeloid leukemia
